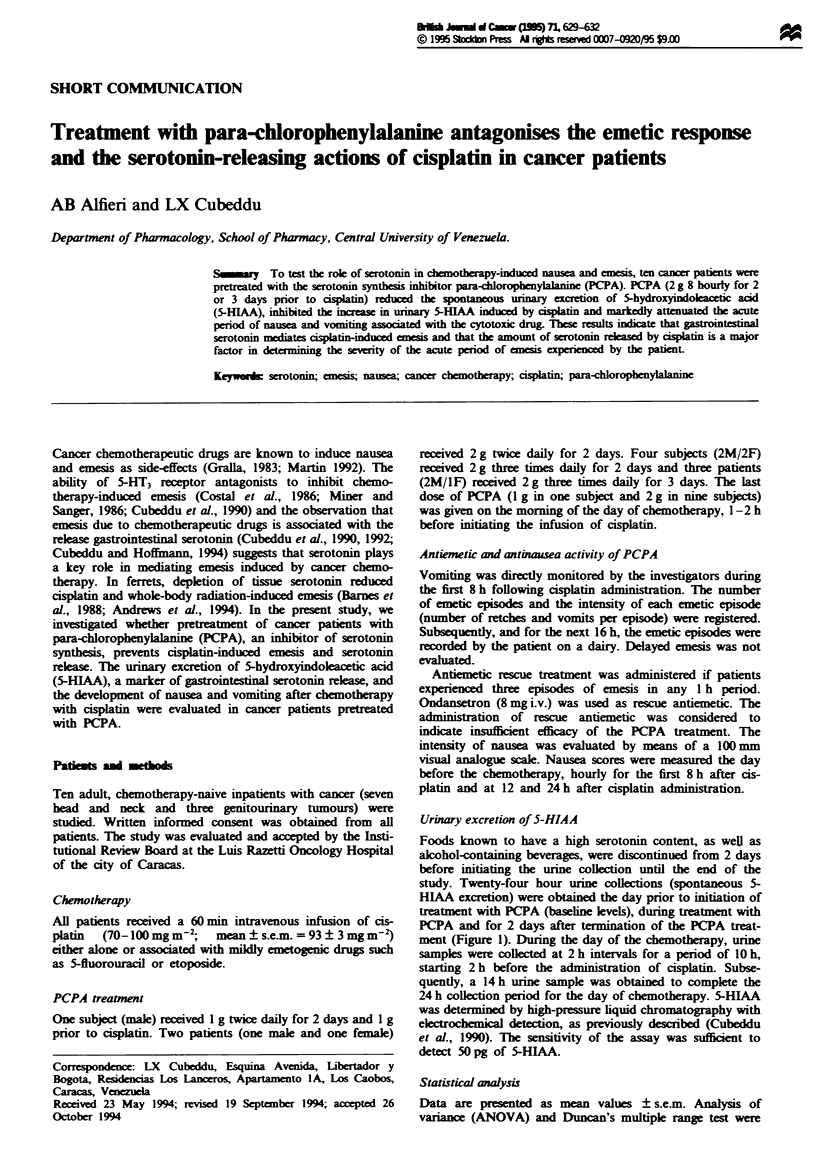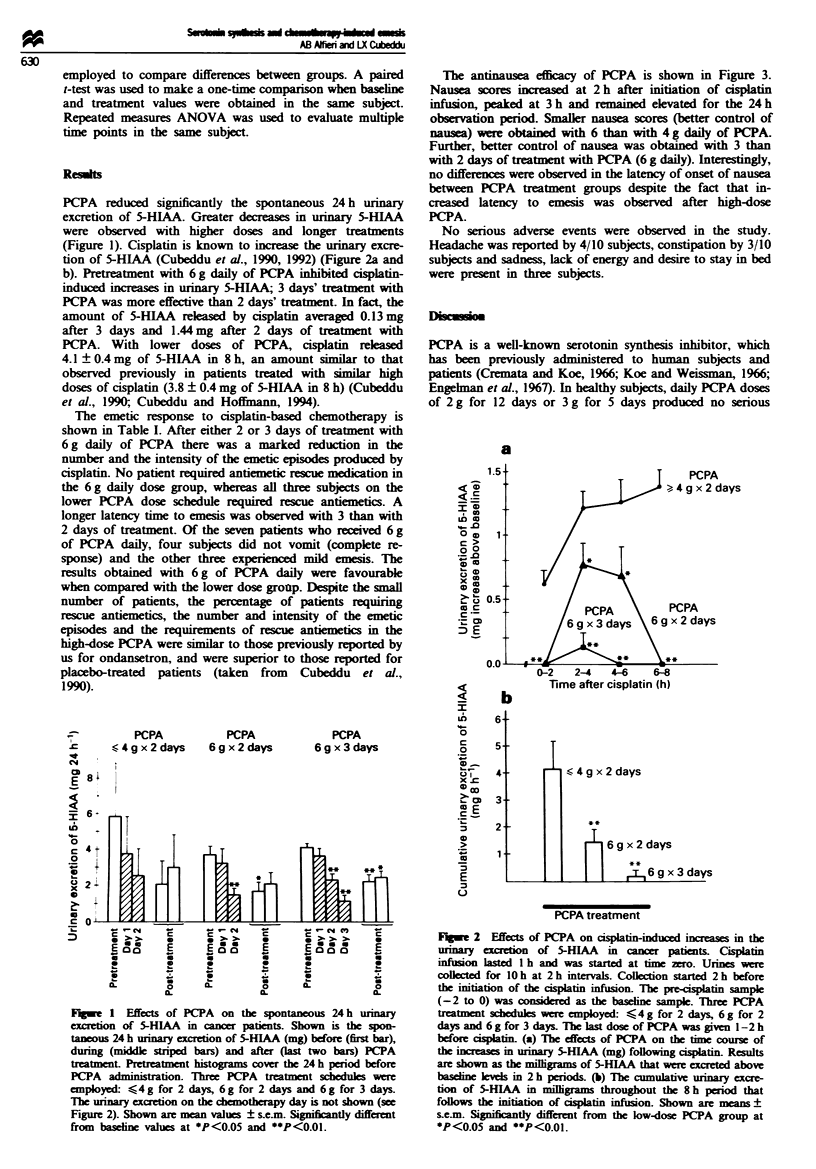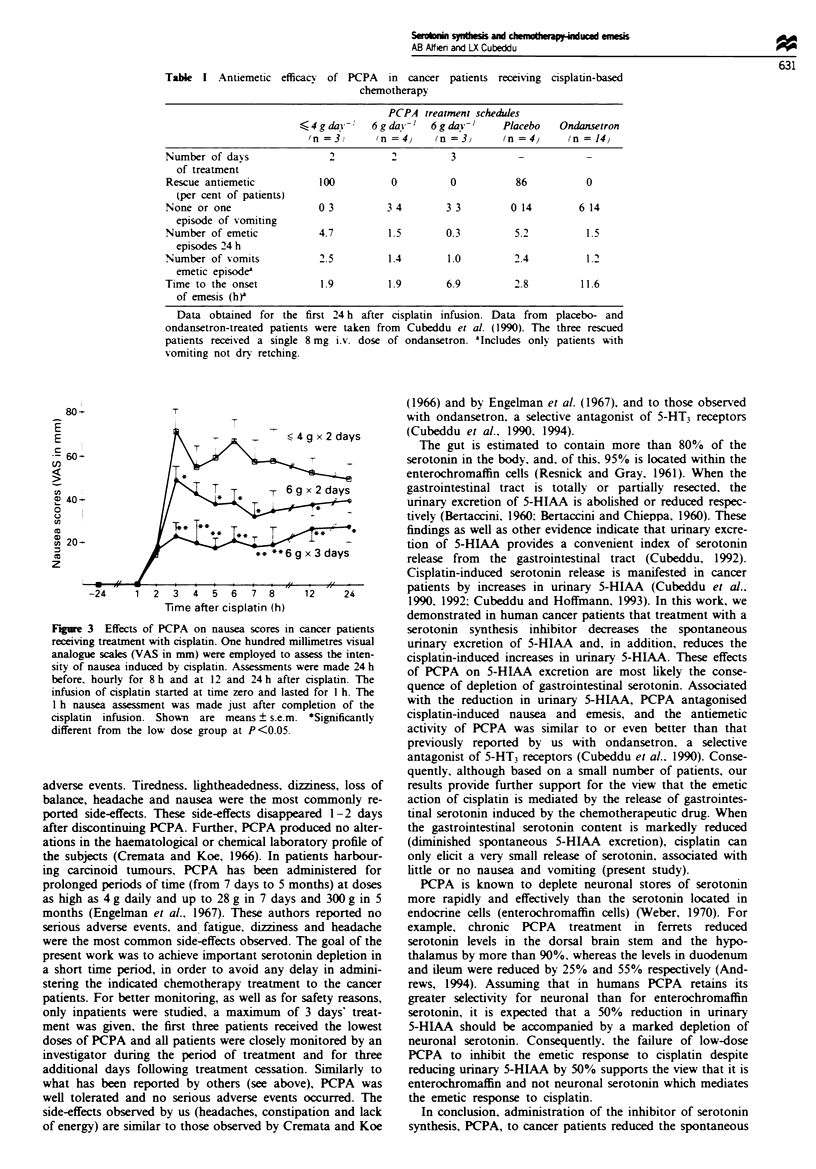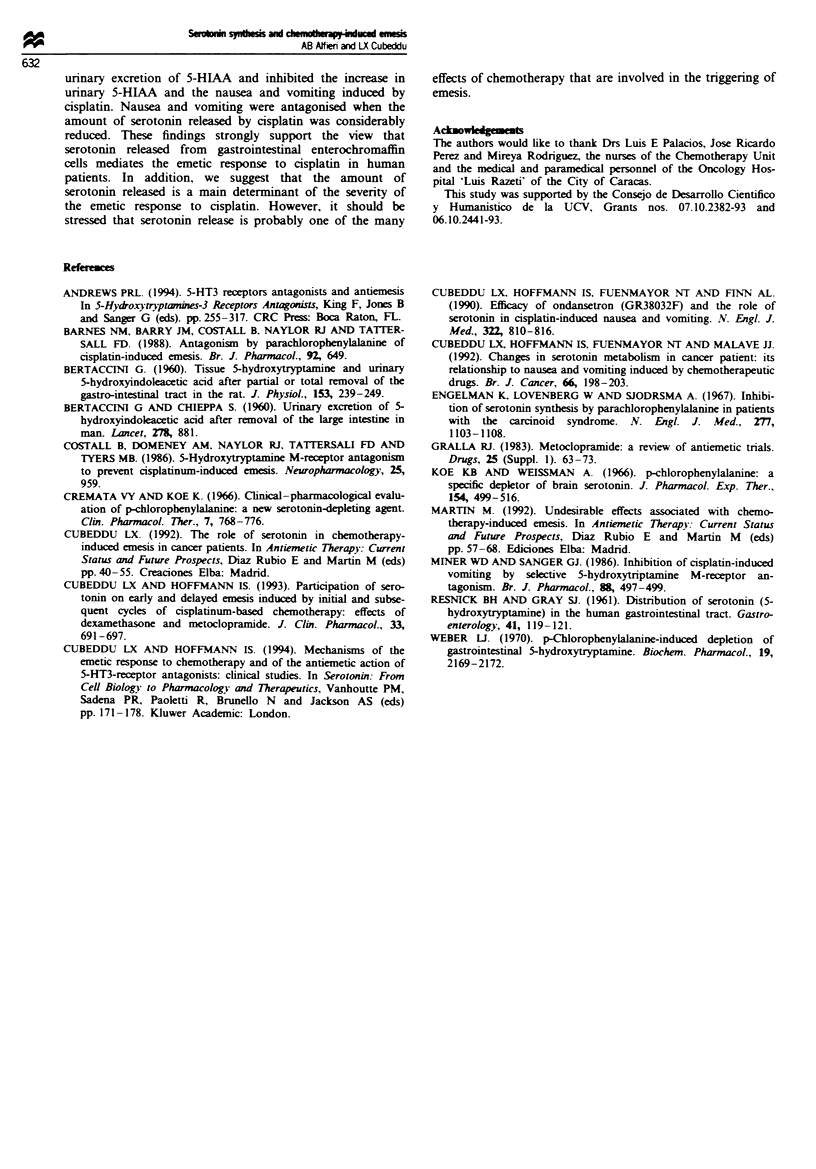# Treatment with para-chlorophenylalanine antagonises the emetic response and the serotonin-releasing actions of cisplatin in cancer patients.

**DOI:** 10.1038/bjc.1995.123

**Published:** 1995-03

**Authors:** A. B. Alfieri, L. X. Cubeddu

**Affiliations:** Department of Pharmacology, School of Pharmacy, Central University of Venezuela.

## Abstract

To test the role of serotonin in chemotherapy-induced nausea and emesis, ten cancer patients were pretreated with the serotonin synthesis inhibitor para-chlorophenylalanine (PCPA). PCPA (2 g 8 hourly for 2 or 3 days prior to cisplatin) reduced the spontaneous urinary excretion of 5-hydroxyindoleacetic acid (5-HIAA), inhibited the increase in urinary 5-HIAA induced by cisplatin and markedly attenuated the acute period of nausea and vomiting associated with the cytotoxic drug. These results indicate that gastrointestinal serotonin mediates cisplatin-induced emesis and that the amount of serotonin released by cisplatin is a major factor in determining the severity of the acute period of emesis experienced by the patient.


© 1995 Stockton Press All rights reserved 0007-0920/95 $9.00

SHORT COMMUNICATION

### Treatment with para-chlorophenylalanine antagonises the emetic response and the serotonin-releasing actions of cisplatin in cancer patients

AB Alfieri and LX Cubeddu

*Department of Pharmacology, School of Pharmacy, Central University of Venezuela.*

**Summary** To test the role of serotonin in chemotherapy-induced nausea and emesis, ten cancer patients were pretreated with the serotonin synthesis inhibitor para-chlorophenylalanine (PCPA). PCPA (2 g 8 hourly for 2 or 3 days prior to cisplatin) reduced the spontaneous urinary excretion of 5-hydroxyindoleacetic acid (5-HIAA), inhibited the increase in urinary 5-HIAA induced by cisplatin and markedly attenuated the acute period of nausea and vomiting associated with the cytotoxic drug. These results indicate that gastrointestinal serotonin mediates cisplatin-induced emesis and that the amount of serotonin released by cisplatin is a major factor in determining the severity of the acute period of emesis experienced by the patient.

**Keywords:** serotonin; emesis; nausea; cancer chemotherapy; cisplatin; para-chlorophenylalanine

Cancer chemotherapeutic drugs are known to induce nausea and emesis as side-effects (Gralla, 1983; Martin 1992). The ability of 5-$HT_3$ receptor antagonists to inhibit chemotherapy-induced emesis (Costal *et al.*, 1986; Miner and Sanger, 1986; Cubeddu *et al.*, 1990) and the observation that emesis due to chemotherapeutic drugs is associated with the release gastrointestinal serotonin (Cubeddu *et al.*, 1990, 1992; Cubeddu and Hoffmann, 1994) suggests that serotonin plays a key role in mediating emesis induced by cancer chemotherapy. In ferrets, depletion of tissue serotonin reduced cisplatin and whole-body radiation-induced emesis (Barnes *et al.*, 1988; Andrews *et al.*, 1994). In the present study, we investigated whether pretreatment of cancer patients with para-chlorophenylalanine (PCPA), an inhibitor of serotonin synthesis, prevents cisplatin-induced emesis and serotonin release. The urinary excretion of 5-hydroxyindoleacetic acid (5-HIAA), a marker of gastrointestinal serotonin release, and the development of nausea and vomiting after chemotherapy with cisplatin were evaluated in cancer patients pretreated with PCPA.

### Patients and methods

Ten adult, chemotherapy-naive inpatients with cancer (seven head and neck and three genitourinary tumours) were studied. Written informed consent was obtained from all patients. The study was evaluated and accepted by the Institutional Review Board at the Luis Razetti Oncology Hospital of the city of Caracas.

### *Chemotherapy*

All patients received a 60 min intravenous infusion of cisplatin (70-100 mg $m^{-2}$; mean ± s.e.m. = 93 ± 3 mg $m^{-2}$) either alone or associated with mildly emetogenic drugs such as 5-fluorouracil or etoposide.

### *PCPA treatment*

One subject (male) received 1 g twice daily for 2 days and 1 g prior to cisplatin. Two patients (one male and one female) received 2 g twice daily for 2 days. Four subjects (2M/2F) received 2 g three times daily for 2 days and three patients (2M/1F) received 2 g three times daily for 3 days. The last dose of PCPA (1 g in one subject and 2 g in nine subjects) was given on the morning of the day of chemotherapy, 1-2 h before initiating the infusion of cisplatin.

### *Antiemetic and antinausea activity of PCPA*

Vomiting was directly monitored by the investigators during the first 8 h following cisplatin administration. The number of emetic episodes and the intensity of each emetic episode (number of retches and vomits per episode) were registered. Subsequently, and for the next 16 h, the emetic episodes were recorded by the patient on a dairy. Delayed emesis was not evaluated.

Antiemetic rescue treatment was administered if patients experienced three episodes of emesis in any 1 h period. Ondansetron (8 mg i.v.) was used as rescue antiemetic. The administration of rescue antiemetic was considered to indicate insufficient efficacy of the PCPA treatment. The intensity of nausea was evaluated by means of a 100 mm visual analogue scale. Nausea scores were measured the day before the chemotherapy, hourly for the first 8 h after cisplatin and at 12 and 24 h after cisplatin administration.

### *Urinary excretion of 5-HIAA*

Foods known to have a high serotonin content, as well as alcohol-containing beverages, were discontinued from 2 days before initiating the urine collection until the end of the study. Twenty-four hour urine collections (spontaneous 5-HIAA excretion) were obtained the day prior to initiation of treatment with PCPA (baseline levels), during treatment with PCPA and for 2 days after termination of the PCPA treatment (Figure 1). During the day of the chemotherapy, urine samples were collected at 2 h intervals for a period of 10 h, starting 2 h before the administration of cisplatin. Subsequently, a 14 h urine sample was obtained to complete the 24 h collection period for the day of chemotherapy. 5-HIAA was determined by high-pressure liquid chromatography with electrochemical detection, as previously described (Cubeddu *et al.*, 1990). The sensitivity of the assay was sufficient to detect 50 pg of 5-HIAA.

### *Statistical analysis*

Data are presented as mean values ± s.e.m. Analysis of variance (ANOVA) and Duncan's multiple range test were

Correspondence: LX Cubeddu, Esquina Avenida, Libertador y Bogota, Residencias Los Lanceros, Apartamento 1A, Los Caobos, Caracas, Venezuela
Received 23 May 1994; revised 19 September 1994; accepted 26 October 1994

employed to compare differences between groups. A paired $t$-test was used to make a one-time comparison when baseline and treatment values were obtained in the same subject. Repeated measures ANOVA was used to evaluate multiple time points in the same subject.

### Results

PCPA reduced significantly the spontaneous 24 h urinary excretion of 5-HIAA. Greater decreases in urinary 5-HIAA were observed with higher doses and longer treatments (Figure 1). Cisplatin is known to increase the urinary excretion of 5-HIAA (Cubeddu *et al.*, 1990, 1992) (Figure 2a and b). Pretreatment with 6 g daily of PCPA inhibited cisplatin-induced increases in urinary 5-HIAA; 3 days' treatment with PCPA was more effective than 2 days' treatment. In fact, the amount of 5-HIAA released by cisplatin averaged 0.13 mg after 3 days and 1.44 mg after 2 days of treatment with PCPA. With lower doses of PCPA, cisplatin released 4.1 ± 0.4 mg of 5-HIAA in 8 h, an amount similar to that observed previously in patients treated with similar high doses of cisplatin (3.8 ± 0.4 mg of 5-HIAA in 8 h) (Cubeddu *et al.*, 1990; Cubeddu and Hoffmann, 1994).

The emetic response to cisplatin-based chemotherapy is shown in Table I. After either 2 or 3 days of treatment with 6 g daily of PCPA there was a marked reduction in the number and the intensity of the emetic episodes produced by cisplatin. No patient required antiemetic rescue medication in the 6 g daily dose group, whereas all three subjects on the lower PCPA dose schedule required rescue antiemetics. A longer latency time to emesis was observed with 3 than with 2 days of treatment. Of the seven patients who received 6 g of PCPA daily, four subjects did not vomit (complete response) and the other three experienced mild emesis. The results obtained with 6 g of PCPA daily were favourable when compared with the lower dose group. Despite the small number of patients, the percentage of patients requiring rescue antiemetics, the number and intensity of the emetic episodes and the requirements of rescue antiemetics in the high-dose PCPA were similar to those previously reported by us for ondansetron, and were superior to those reported for placebo-treated patients (taken from Cubeddu *et al.*, 1990).

**Figure 1** Effects of PCPA on the spontaneous 24 h urinary excretion of 5-HIAA in cancer patients. Shown is the spontaneous 24 h urinary excretion of 5-HIAA (mg) before (first bar), during (middle striped bars) and after (last two bars) PCPA treatment. Pretreatment histograms cover the 24 h period before PCPA administration. Three PCPA treatment schedules were employed: ≤4 g for 2 days, 6 g for 2 days and 6 g for 3 days. The urinary excretion on the chemotherapy day is not shown (see Figure 2). Shown are mean values ± s.e.m. Significantly different from baseline values at *$P$<0.05 and **$P$<0.01.

The antinausea efficacy of PCPA is shown in Figure 3. Nausea scores increased at 2 h after initiation of cisplatin infusion, peaked at 3 h and remained elevated for the 24 h observation period. Smaller nausea scores (better control of nausea) were obtained with 6 than with 4 g daily of PCPA. Further, better control of nausea was obtained with 3 than with 2 days of treatment with PCPA (6 g daily). Interestingly, no differences were observed in the latency of onset of nausea between PCPA treatment groups despite the fact that increased latency to emesis was observed after high-dose PCPA.

No serious adverse events were observed in the study. Headache was reported by 4/10 subjects, constipation by 3/10 subjects and sadness, lack of energy and desire to stay in bed were present in three subjects.

### Discussion

PCPA is a well-known serotonin synthesis inhibitor, which has been previously administered to human subjects and patients (Cremata and Koe, 1966; Koe and Weissman, 1966; Engelman *et al.*, 1967). In healthy subjects, daily PCPA doses of 2 g for 12 days or 3 g for 5 days produced no serious

**Figure 2** Effects of PCPA on cisplatin-induced increases in the urinary excretion of 5-HIAA in cancer patients. Cisplatin infusion lasted 1 h and was started at time zero. Urines were collected for 10 h at 2 h intervals. Collection started 2 h before the initiation of the cisplatin infusion. The pre-cisplatin sample (−2 to 0) was considered as the baseline sample. Three PCPA treatment schedules were employed: ≤4 g for 2 days, 6 g for 2 days and 6 g for 3 days. The last dose of PCPA was given 1–2 h before cisplatin. (**a**) The effects of PCPA on the time course of the increases in urinary 5-HIAA (mg) following cisplatin. Results are shown as the milligrams of 5-HIAA that were excreted above baseline levels in 2 h periods. (**b**) The cumulative urinary excretion of 5-HIAA in milligrams throughout the 8 h period that follows the initiation of cisplatin infusion. Shown are means ± s.e.m. Significantly different from the low-dose PCPA group at *$P$<0.05 and **$P$<0.01.

**Table I** Antiemetic efficacy of PCPA in cancer patients receiving cisplatin-based chemotherapy

| | *PCPA treatment schedules* | | | | |
|---|---|---|---|---|---|
| | ≤ *4 g day*$^{-1}$ (n = *3*) | *6 g day*$^{-1}$ (n = *4*) | *6 g day*$^{-1}$ (n = *3*) | *Placebo* (n = *4*) | *Ondansetron* (n = *14*) |
| Number of days of treatment | 2 | 2 | 3 | – | – |
| Rescue antiemetic (per cent of patients) | 100 | 0 | 0 | 86 | 0 |
| None or one episode of vomiting | 0 3 | 3 4 | 3 3 | 0 14 | 6 14 |
| Number of emetic episodes 24 h | 4.7 | 1.5 | 0.3 | 5.2 | 1.5 |
| Number of vomits emetic episode[a] | 2.5 | 1.4 | 1.0 | 2.4 | 1.2 |
| Time to the onset of emesis (h)[a] | 1.9 | 1.9 | 6.9 | 2.8 | 11.6 |

Data obtained for the first 24 h after cisplatin infusion. Data from placebo- and ondansetron-treated patients were taken from Cubeddu *et al.* (1990). The three rescued patients received a single 8 mg i.v. dose of ondansetron. [a]Includes only patients with vomiting not dry retching.

**Figure 3** Effects of PCPA on nausea scores in cancer patients receiving treatment with cisplatin. One hundred millimetres visual analogue scales (VAS in mm) were employed to assess the intensity of nausea induced by cisplatin. Assessments were made 24 h before, hourly for 8 h and at 12 and 24 h after cisplatin. The infusion of cisplatin started at time zero and lasted for 1 h. The 1 h nausea assessment was made just after completion of the cisplatin infusion. Shown are means ± s.e.m. *Significantly different from the low dose group at $P<0.05$.

adverse events. Tiredness, lightheadedness, dizziness, loss of balance, headache and nausea were the most commonly reported side-effects. These side-effects disappeared 1–2 days after discontinuing PCPA. Further, PCPA produced no alterations in the haematological or chemical laboratory profile of the subjects (Cremata and Koe, 1966). In patients harbouring carcinoid tumours, PCPA has been administered for prolonged periods of time (from 7 days to 5 months) at doses as high as 4 g daily and up to 28 g in 7 days and 300 g in 5 months (Engelman *et al.*, 1967). These authors reported no serious adverse events, and fatigue, dizziness and headache were the most common side-effects observed. The goal of the present work was to achieve important serotonin depletion in a short time period, in order to avoid any delay in administering the indicated chemotherapy treatment to the cancer patients. For better monitoring, as well as for safety reasons, only inpatients were studied, a maximum of 3 days' treatment was given, the first three patients received the lowest doses of PCPA and all patients were closely monitored by an investigator during the period of treatment and for three additional days following treatment cessation. Similarly to what has been reported by others (see above), PCPA was well tolerated and no serious adverse events occurred. The side-effects observed by us (headaches, constipation and lack of energy) are similar to those observed by Cremata and Koe (1966) and by Engelman *et al.* (1967), and to those observed with ondansetron, a selective antagonist of 5-$HT_3$ receptors (Cubeddu *et al.*, 1990, 1994).

The gut is estimated to contain more than 80% of the serotonin in the body, and, of this, 95% is located within the enterochromaffin cells (Resnick and Gray, 1961). When the gastrointestinal tract is totally or partially resected, the urinary excretion of 5-HIAA is abolished or reduced respectively (Bertaccini, 1960; Bertaccini and Chieppa, 1960). These findings as well as other evidence indicate that urinary excretion of 5-HIAA provides a convenient index of serotonin release from the gastrointestinal tract (Cubeddu, 1992). Cisplatin-induced serotonin release is manifested in cancer patients by increases in urinary 5-HIAA (Cubeddu *et al.*, 1990, 1992; Cubeddu and Hoffmann, 1993). In this work, we demonstrated in human cancer patients that treatment with a serotonin synthesis inhibitor decreases the spontaneous urinary excretion of 5-HIAA and, in addition, reduces the cisplatin-induced increases in urinary 5-HIAA. These effects of PCPA on 5-HIAA excretion are most likely the consequence of depletion of gastrointestinal serotonin. Associated with the reduction in urinary 5-HIAA, PCPA antagonised cisplatin-induced nausea and emesis, and the antiemetic activity of PCPA was similar to or even better than that previously reported by us with ondansetron, a selective antagonist of 5-$HT_3$ receptors (Cubeddu *et al.*, 1990). Consequently, although based on a small number of patients, our results provide further support for the view that the emetic action of cisplatin is mediated by the release of gastrointestinal serotonin induced by the chemotherapeutic drug. When the gastrointestinal serotonin content is markedly reduced (diminished spontaneous 5-HIAA excretion), cisplatin can only elicit a very small release of serotonin, associated with little or no nausea and vomiting (present study).

PCPA is known to deplete neuronal stores of serotonin more rapidly and effectively than the serotonin located in endocrine cells (enterochromaffin cells) (Weber, 1970). For example, chronic PCPA treatment in ferrets reduced serotonin levels in the dorsal brain stem and the hypothalamus by more than 90%, whereas the levels in duodenum and ileum were reduced by 25% and 55% respectively (Andrews, 1994). Assuming that in humans PCPA retains its greater selectivity for neuronal than for enterochromaffin serotonin, it is expected that a 50% reduction in urinary 5-HIAA should be accompanied by a marked depletion of neuronal serotonin. Consequently, the failure of low-dose PCPA to inhibit the emetic response to cisplatin despite reducing urinary 5-HIAA by 50% supports the view that it is enterochromaffin and not neuronal serotonin which mediates the emetic response to cisplatin.

In conclusion, administration of the inhibitor of serotonin synthesis, PCPA, to cancer patients reduced the spontaneous urinary excretion of 5-HIAA and inhibited the increase in urinary 5-HIAA and the nausea and vomiting induced by cisplatin. Nausea and vomiting were antagonised when the amount of serotonin released by cisplatin was considerably reduced. These findings strongly support the view that serotonin released from gastrointestinal enterochromaffin cells mediates the emetic response to cisplatin in human patients. In addition, we suggest that the amount of serotonin released is a main determinant of the severity of the emetic response to cisplatin. However, it should be stressed that serotonin release is probably one of the many effects of chemotherapy that are involved in the triggering of emesis.

**Acknowledgements**

The authors would like to thank Drs Luis E Palacios, Jose Ricardo Perez and Mireya Rodriguez, the nurses of the Chemotherapy Unit and the medical and paramedical personnel of the Oncology Hospital 'Luis Razeti' of the City of Caracas.

This study was supported by the Consejo de Desarrollo Cientifico y Humanistico de la UCV, Grants nos. 07.10.2382-93 and 06.10.2441-93.

### References

ANDREWS PRL. (1994). 5-HT3 receptors antagonists and antiemesis In *5-Hydroxytryptamines-3 Receptors Antagonists*, King F, Jones B and Sanger G (eds). pp. 255-317. CRC Press: Boca Raton, FL.

BARNES NM, BARRY JM, COSTALL B, NAYLOR RJ AND TATTERSALL FD. (1988). Antagonism by parachlorophenylalanine of cisplatin-induced emesis. *Br. J. Pharmacol.*, **92**, 649.

BERTACCINI G. (1960). Tissue 5-hydroxytryptamine and urinary 5-hydroxyindoleacetic acid after partial or total removal of the gastro-intestinal tract in the rat. *J. Physiol.*, **153**, 239-249.

BERTACCINI G AND CHIEPPA S. (1960). Urinary excretion of 5-hydroxyindoleacetic acid after removal of the large intestine in man. *Lancet*, **278**, 881.

COSTALL B, DOMENEY AM, NAYLOR RJ, TATTERSALI FD AND TYERS MB. (1986). 5-Hydroxytryptamine M-receptor antagonism to prevent cisplatinum-induced emesis. *Neuropharmacology*, **25**, 959.

CREMATA VY AND KOE K. (1966). Clinical-pharmacological evaluation of p-chlorophenylalanine: a new serotonin-depleting agent. *Clin. Pharmacol. Ther.*, **7**, 768-776.

CUBEDDU LX. (1992). The role of serotonin in chemotherapy-induced emesis in cancer patients. In *Antiemetic Therapy: Current Status and Future Prospects*, Diaz Rubio E and Martin M (eds) pp. 40-55. Creaciones Elba: Madrid.

CUBEDDU LX AND HOFFMANN IS. (1993). Participation of serotonin on early and delayed emesis induced by initial and subsequent cycles of cisplatinum-based chemotherapy: effects of dexamethasone and metoclopramide. *J. Clin. Pharmacol.*, **33**, 691-697.

CUBEDDU LX AND HOFFMANN IS. (1994). Mechanisms of the emetic response to chemotherapy and of the antiemetic action of 5-HT3-receptor antagonists: clinical studies. In *Serotonin: From Cell Biology to Pharmacology and Therapeutics*, Vanhoutte PM, Sadena PR, Paoletti R, Brunello N and Jackson AS (eds) pp. 171-178. Kluwer Academic: London.

CUBEDDU LX, HOFFMANN IS, FUENMAYOR NT AND FINN AL. (1990). Efficacy of ondansetron (GR38032F) and the role of serotonin in cisplatin-induced nausea and vomiting. *N. Engl. J. Med.*, **322**, 810-816.

CUBEDDU LX, HOFFMANN IS, FUENMAYOR NT AND MALAVE JJ. (1992). Changes in serotonin metabolism in cancer patient: its relationship to nausea and vomiting induced by chemotherapeutic drugs. *Br. J. Cancer*, **66**, 198-203.

ENGELMAN K, LOVENBERG W AND SJODRSMA A. (1967). Inhibition of serotonin synthesis by parachlorophenylalanine in patients with the carcinoid syndrome. *N. Engl. J. Med.*, **277**, 1103-1108.

GRALLA RJ. (1983). Metoclopramide: a review of antiemetic trials. *Drugs*, **25** (Suppl. 1). 63-73.

KOE KB AND WEISSMAN A. (1966). p-chlorophenylalanine: a specific depletor of brain serotonin. *J. Pharmacol. Exp. Ther.*, **154**, 499-516.

MARTIN M. (1992). Undesirable effects associated with chemotherapy-induced emesis. In *Antiemetic Therapy: Current Status and Future Prospects*, Diaz Rubio E and Martin M (eds) pp. 57-68. Ediciones Elba: Madrid.

MINER WD AND SANGER GJ. (1986). Inhibition of cisplatin-induced vomiting by selective 5-hydroxytriptamine M-receptor antagonism. *Br. J. Pharmacol.*, **88**, 497-499.

RESNICK BH AND GRAY SJ. (1961). Distribution of serotonin (5-hydroxytryptamine) in the human gastrointestinal tract. *Gastroenterology*, **41**, 119-121.

WEBER LJ. (1970). p-Chlorophenylalanine-induced depletion of gastrointestinal 5-hydroxytryptamine. *Biochem. Pharmacol.*, **19**, 2169-2172.